# Elevation of Highly Sensitive Cardiac Troponin T Among End-Stage Renal Disease Patients Without Acute Coronary Syndrome

**DOI:** 10.21315/mjms2021.28.5.6

**Published:** 2021-10-26

**Authors:** Wan Mohd Zamri Wan Nur Aimi, Shafii Noorazliyana, Tuan Ismail Tuan Salwani, Zakaria Adlin Zafrulan, Yaacob Najib Majdi, Che Soh Noor Azlin Azraini

**Affiliations:** 1 Department of Pathology, Hospital Selayang, Batu Caves, Selangor, Malaysia; 2 Department of Chemical Pathology, School of Medical Sciences, Universiti Sains Malaysia, Kubang Kerian, Kelantan, Malaysia; 3 Department of Pathology, Hospital Raja Perempuan Zainab II, Kota Bharu, Kelantan, Malaysia; 4 Unit of Biostatistics and Research Methodology, School of Medical Sciences, Universiti Sains Malaysia, Kubang Kerian, Kelantan, Malaysia

**Keywords:** highly sensitive cardiac troponin T, end-stage renal disease, acute coronary syndrome

## Abstract

**Background:**

In end-stage renal disease (ESRD), troponin T concentrations can be elevated even without cardiac ischaemia, which hampers the diagnosis of acute myocardial infarction (AMI). The objectives of our study were to determine the proportion of dialysisdependent ESRD patients without acute coronary syndrome (ACS) but with highly sensitive cardiac troponin T (hs-cTnT) levels above the 99th percentile upper reference limit and to evaluate the range of hs-cTnT among this population.

**Methods:**

A cross-sectional study was conducted at the haemodialysis (HD) unit of a tertiary hospital in Malaysia from January 2018 to February 2019. Dialysis-dependent ESRD patients were included and those with a recent history of ACS (within 30 days) were excluded. Pre-dialysed serum hs-cTnT levels were measured using Cobas e411. The upper limit of the 99th percentile value for troponin T was 14 ng/L.

**Results:**

A total of 150 patients were recruited as study participants. The majority were female (62%) and of Malay ethnicity (94%), and the mean (SD) age was 45.19 (16.36) years old. The hs-cTnT range (min, max) was 11.39–738.30 ng/L and the median (interquartile range [IQR]) of hs-cTnT was 59.20 (83.41) ng/L. Elevated hs-cTnT levels were observed in 149/150 (99%) of the study participants (54/55 [98.2%] of the patients were on HD, and 95/95 [100.0%] of the patients were on continuous ambulatory peritoneal dialysis).

**Conclusion:**

This study supports prior research showing that even without ACS, most ESRD patients have elevated concentrations of cardiac troponin. Furthermore, our study illustrates the need to revisit the use of absolute troponin values when making a diagnosis of ACS in ESRD patients.

## Introduction

For the past 30 years, cardiac troponin (cTn) levels have been used as the diagnostic biochemical marker for pathologies associated with cardiomyocyte death, particularly myocardial infarction (1). Serial measurements of cTn values as well as other elements of the clinical evaluation are needed to establish a diagnosis of acute myocardial infarction (AMI) (2–3). Troponins are regulatory protein complexes located on the thin filaments of striated muscles and consist of three different subunits: troponin C, troponin I and troponin T. They control the interaction of calcium-mediated actin and myosin for muscle contraction (1). Troponins are expressed in both cardiac and skeletal muscles.

Cardiac troponin T (cTnT) is a low molecular weight protein weighing 37 kDa (4) and is encoded by different genes to those of skeletal muscle troponin (1). cTnT is expressed in cardiomyocytes; thus, it is structurally specific to the myocardium compared to creatine kinase or creatine kinase-MB (1, 5). The presence of cTnT in peripheral blood is indicative of cardiomyocyte damage.

Cardiac-like TnT isoforms are found in human foetal skeletal muscle but disappear as humans mature (6). However, troponin I (cTnI) is more cardiac-specific and is not expressed in skeletal muscles and other tissues during foetal development or in degenerative/regenerative muscle diseases (7). While both cTnT and cTnI are organ-specific, they are not disease-specific as both can be released as a result of ischaemic, non-ischaemic and extra-cardiac conditions (3, 8, 9).

Even without AMI, end-stage renal disease (ESRD) patients are known to have a greater incidence of chronically elevated cTn compared to non-ESRD patients (10–12). No single mechanism explains the chronic elevation of cTn in ESRD patients. It was previously believed to be due to uraemic myopathy syndrome (13–14). Uraemia and metabolic perturbations of renal failure may stimulate the re-expression of these cardiac-like TnT from the skeletal muscles (13). However, with the assay advancement of cardiac isoform specificity over that of skeletal isoforms, this appears less likely to be the cause.

The highly sensitive troponin T (hs-cTnT) assay has a reported 0.003% cross-reactivity with human skeletal muscle troponin T (Roche Diagnostics GmbH, Troponin T hs STAT 2013). The uraemic state also has a direct toxic effect on the apoptosis of cardiomyocytes (15). In dialysis-dependent patients, recurrent cardiomyocyte damage from dialysis-related myocardial stunning is one possible explanation for the chronicity of elevated cTnT concentrations (16). It is widely believed that reduced renal clearance contributes to the high cTn values found in ESRD patients (17–18). This fact is further supported by the decline of cTn levels in patients following a kidney transplant (19). However, another study has disputed renal clearance theory as a cause of elevated cTn values. Ellis et al. (20) did not observe a statistically significant difference in the half-life or the elimination rate constant of cTnI in patients with AMI and ESRD compared to patients with AMI and normal kidney function.

In the absence of AMI, direct cardiac damage via, for example, silent micro-infarcts (13), myocardial strain and oxygen mismatch from high left ventricular (LV) preload and afterload (21–22) may be the causes of elevated cTn. However, silent micro-infarcts may not be recognised clinically and ESRD patients may suffer repeated occult ischaemic injury episodes (10, 13). Other mechanisms that have been evaluated are anaemias (23), oxidative stress (24) and increased inflammation (25). To conclude, the exact mechanisms of chronically elevated cTn concentrations remain highly controversial and poorly understood.

With high cardiovascular morbidity and mortality among ESRD patients, clinicians must diagnose and manage elevated cTn accurately. Nevertheless, this can be particularly challenging. Electrocardiography (ECG) findings are frequently abnormal and non-conclusive due to the high prevalence of LV hypertrophy and electrolyte imbalances. Chronically elevated cTn levels in ESRD patients and the complexity of the presenting symptoms reduce the specificity of cTn for diagnosing AMI. To make matters more challenging, with improvement in assay specificity, such as in hs-cTn assays, most ESRD patients will have elevated cTn levels above the 99th percentile upper reference limit (URL) (26). Using this cut-off value is therefore problematic in diagnosing ESRD patients with suspected ACS (5, 27–30).

This study was conducted to investigate the proportion of ESRD patients without ACS but with elevated hs-cTnT levels above the 99th percentile URL and to provide knowledge on the ranges of hs-cTnT values among dialysis-dependent ESRD patients without ACS.

## Methods

This cross-sectional study was conducted at the Haemodialysis Unit of Hospital Raja Perempuan Zainab II (HRPZ II), Kota Bharu, Kelantan Malaysia, between January 2018 and February 2019.

The eligibility criteria were all in-centre ESRD patients, defined as those having a glomerular filtration rate (GFR) of less than 15 mL/min/1.73 m^2^ based on the Modification of Diet in Renal Disease GFR equation, patients aged 18 years old and above, patients on haemodialysis (HD) or continuous ambulatory peritoneal dialysis (CAPD) and patients without ACS or ECG changes suggestive of MI during the 30-day period prior to recruitment. The exclusion criteria were medical conditions that cause elevated cTnT levels (e.g. post percutaneous coronary intervention, open-heart surgery, acute pulmonary embolism, pericarditis/myocarditis, aortic dissection, heart failure, cardiotoxic drugs [Herceptin, anthracyclines], cardiac contusion/ablation/pacing/defibrillation shock, rhabdomyolysis or infiltrative cardiac diseases) (3, 31), a history of hospitalisation for any cause within the 30 days prior to screening, patients with septicaemia or those who were critically ill, ECG changes suggestive of myocardial infarction or ECG changes suggestive of medical conditions other than the aforementioned. All the exclusion criteria were assessed based on the patients’ histories and clinical examinations and a review of their medical records.

No sampling method was applied as all the eligible patients were included in this study. The ESRD patients were screened and selected based on the inclusion and exclusion criteria. Of the 184 patients screened for eligibility, 33 were not eligible and one patient refused to participate. A total of 150 patients were therefore recruited. Demographic data, the aetiology of ESRD, the type of dialysis and the duration of dialysis were obtained from the dialysis unit’s medical records. For each patient recruited, possible ACS symptoms were assessed before a 12-lead ECG was performed and interpreted based on the Fourth Universal Definition of Myocardial Infarction (2018) document (3).

A volume of 2.5 mL–3.0 mL pre-dialysis venous blood was collected in a gel separator tube. The samples were centrifuged and stored in a freezer at −20°C at the Chemical Pathology Laboratory at HRPZ II and later transported (while maintaining a temperature of 2°C–8 °C) to the Chemical Pathology Laboratory Hospital Universiti Sains Malaysia for hs-cTnT analysis. The samples with a pre-analytical error, such as haemolysed samples, were excluded.

The serum for cTnT analysis was tested using the hs-cTnT Cobas e411 Analyser (electrochemiluminescence immunoassay method; Roche Diagnostics, Indianapolis, USA). The analytical characteristics of highly sensitive assays should demonstrate a total imprecision or coefficient variation (CV) of ≤ 10% at the 99th percentile of the population of interest and the assay’s ability to quantitate at least 50% of healthy individuals of the population of interest (32).

According to the manufacturer’s assay, the upper limit of the 99th percentile value for hs-cTnT is 14 ng/L with a 10% CV value of 13.0 ng/L. A value above 14 ng/L was thus considered abnormal. The limit of detection was 5 ng/L and the analytical range was 3 ng/L–10,000 ng/L. The sensitivity and specificity of the hs-cTnT calculated at the 99th percentile of 14 ng/L were 100% and 75%, respectively.

The data analysis was performed using IBM SPSS software version 24 (Chicago, USA). The categorical variables were reported as frequency and percentage, while the numerical variables were described as mean and standard deviation (SD) as they were normally distributed. This was determined using a test of normality (Kolmogorov–Smirnov test) and a histogram with an overlaid normal curve.

## Results

The baseline characteristics of the study participants are summarised in Table 1. The aetiologies of ESRD comprised five patients with an undetermined/unknown aetiology, three patients with obstructive uropathy, two patients with the atypical haemolytic uraemic syndrome, one patient with adult polycystic kidney disease and one patient with congenital posterior urethral valve.

The hs-cTnT results were positively skewed with non-Gaussian distribution, as confirmed by a one-sample Lilliefors-corrected Kolmogorov-Smirnov (K-S) test of normality (K-S distance = 0.257, *P* < 0.05). The observed minimum and maximum values of hs-cTnT were 11.39 ng/L and 738.30 ng/L, respectively.

Table 2 shows the hs-cTnT values among the HD and CAPD patients and the proportion of CAPD and HD patients with hs-cTnT values below and above the 99th percentile URL of hs-cTnT.

## Discussion

The hs-cTnT concentrations in patients dialysis-dependent ESRD are known to be higher than the normal reference population cut-off, even without AMI (26, 30, 33). This chronic elevation of cTnT in ESRD makes the diagnosis of ACS challenging as no data regarding optimal clinical decision levels for hs-cTnT are available. In this study, 99% of the participants had hs-cTnT levels above the cut-off, and among them, 98.2% and 100% were HD and CAPD patients, respectively.

Our finding on the proportion of study participants with elevated cTnT levels is in agreement with that of Artunc et al. (34), who reported that 95% of their study participants had hs-cTnT values above 14 ng/L. Fahim et al. (35) and Keller et al. (36) reported that 90% of their study participants had hs-cTnT concentrations above 14 ng/L, which is slightly lower than our finding. Fahim et al. (35) conducted their study among HD and peritoneal dialysis patients, whereas Keller et al. (36) included only HD patients in their study. Jacob et al. (37), who used a similar 99th percentile URL to that applied in our study, described 94% of their ESRD study participants on HD as having elevated hs-cTnT levels (37). A study conducted in Thailand found that 60.67% of the study participants had increased hs-cTnT values (38); however, the study used ≥ 16 ng/L as the cut-off for the 99th percentile. Wang et al. (39) also reported that hs-cTnT levels were detectable in only 54.5% of their study participants. Even though they applied a lower detection limit value of 3 ng/L as the cut-off for detectable cTnT instead of its 99th percentile URL of 13.3 ng/L, the proportion of study participants with elevated cTnT was less than that in our study. This discrepancy could be due to the different selection criteria for the study participants. The participants in both the aforementioned studies had chronic kidney disease (CKD) stages 3–5 but were not dialysis-dependent. Similar findings have been reported in a few other studies (40–41), but the study populations of interest were different.

This study also evaluated the minimum and maximum levels of hs-cTnT in dialysis-dependent ESRD patients without ACS. The minimum and maximum range of the hs-cTnT values were 11.39 ng/L and 738.30 ng/L, respectively. Fahim et al. (35) reported minimum and maximum hs-cTnT values of 8 ng/L and 241 ng/L, respectively. The difference could be explained in terms of the eligibility criteria for their study. All the adults were selected if they had been on maintenance dialysis for ≥ 90 days and had had a stable dialysis prescription for ≥ 30 days and a transthoracic echocardiogram ≤ 12 months prior to screening (35). Pianta et al. (30) obtained a similar finding to that of our study in that 99% of their HD patients had pre-dialysed hs-cTnT concentrations above the 99th percentile URL with a maximum value of slightly less than 600 ng/mL.

The majority of our study participants were of Malay ethnicity (94%) and represented the Kelantanese population in which 91.3% have a Malay background (42). With regard to age, 68% of our study participants were younger than 54 years old. This was in line with the 2016 National Renal Registry (NRR) data, which reported that approximately 52% of those receiving dialysis were below 54 years old of age (43). The majority of our study participants were female. The gender distribution in the Kelantan population is equal (42) and the 2016 NRR data showed that males (54.6%) outweigh females in terms of dialysis treatment rates (43). In Malaysia, 65% and 12% of new patients had diabetes or hypertension, respectively, as a primary renal disease in 2016 (43). More than half of our study participants (56.7%) had hypertension (22.7% in combination with diabetes) as the aetiology of ESRD.

In our study, 63.3% of the participants were receiving CAPD, whereas only 10% of the ESRD Malaysian population receive CAPD (43). Most of the studies have thus been focused on HD patients (34, 36–37). The data from the inclusion of a balanced number of HD and CAPD patients in our study may therefore represent both dialysis modality groups in ESRD patients.

This study had a few limitations. The study was conducted at a single centre and the results may therefore not be representative of the general Malaysian population. Multicentres are required to cover a broader population for better inference to the Malaysian population. Various studies have demonstrated that the proportion of patients with elevated hs-cTnT levels differs based on the different stages of kidney failure. A study by Twerenbold et al. (26) examined the relationship of eGFR with cTn and found that the proportion of study participants with elevated hs-cTnT values was greater among the ESRD patients compared to those with CKD stages 3–5, although their cTn concentrations were measured using different assays generation to that applied in our study.

## Conclusion

In this study, almost all the participants had elevated cTn concentrations that were above the cut-off value. Knowledge of the hs-cTnT ranges may serve as a guide for clinicians managing ESRD patients who present with possible ACS. Serial changes and especially absolute changes, are important in assisting the diagnosis of AMI when interpreting hs-cTnT levels in patients with ESRD. Future studies should be conducted in multiple centres to better represent the Malaysian population and should include the different stages of CKD.

## Figures and Tables

**Table 1:** Baseline characteristics of study subjects (*n* = 150)

Variables			*n* (%)
Gender	Male		57 (38.0)
	Female		93 (62.0)
Age (years old)			45.19 (16.36)*
	Age distribution:	18–34	51 (34.0)
		35–54	51 (34.0)
		55–69	39 (26.0)
		70–90	9 (6.0)
Race	Malay		141 (94.0)
	Chinese		8 (5.3)
	Other; Indonesian		1 (0.7)
Type of dialysis	HD		55 (36.7)
	CAPD		95 (63.3)
Duration of dialysis (months)			49.28 (49.5)*
hs-cTnT level (ng/L)			59.2 (74.8)**
Etiology of ESRD	Diabetes mellitus		12 (8.0)
	Hypertension		51 (34.0)
	Diabetes mellitus and hypertension		34 (22.7)
	Lupus nephritis		11 (7.3)
	Glomerulonephritis		23 (15.3)
	NSAIDs nephropathy		2 (1.4)
	Ig A nephropathy		5 (3.3)
	Others		12 (8.0)

Notes:

* mean (SD);

** median (IQR)

**Table 2:** Level of hs-cTnT based on the type of renal replacement therapy

hs-TnT level (ng/L)	*n* (%)	

	HD (*n* = 55)	CAPD (*n* = 95)
< 14	1 (1.8)	0 (0)
≥ 14	54 (98.2)	95 (100)
